# Associations Between Serum Reproductive Hormone Concentrations and Hormonal Receptor Status Among Postmenopausal Chinese Women With Breast Cancer: An Observational Study

**DOI:** 10.3389/fonc.2022.819756

**Published:** 2022-05-30

**Authors:** Chuner Jiang, Peng Wu, Xiangming He, Jianfen Ni, Xiaowen Ding, Xiaohong Xu, Fangzheng Wang, Dehong Zou

**Affiliations:** ^1^ Department of Breast Tumor Surgery, Cancer Hospital of the University of Chinese Academy of Sciences (Zhejiang Cancer Hospital), Hangzhou, China; ^2^ Institute of Cancer and Basic Medicine (ICBM), Chinese Academy of Sciences, Hangzhou, China; ^3^ Pathology Department, Cancer Hospital of the University of Chinese Academy of Sciences (Zhejiang Cancer Hospital), Hangzhou, China; ^4^ Clinic Laboratory, Cancer Hospital of the University of Chinese Academy of Sciences (Zhejiang Cancer Hospital), Hangzhou, China; ^5^ Department of Radiation Oncology, Cancer Hospital of the University of Chinese Academy of Sciences (Zhejiang Cancer Hospital), Hangzhou, China

**Keywords:** breast cancer, postmenopausal, reproductive hormones, estrogen receptor, progesterone receptor, HER2 receptor

## Abstract

**Background and Objectives:**

Reproductive hormones and receptors play crucial roles in breast cancer development and progression. The association between preoperative serum reproductive hormone levels and receptor status in postmenopausal women with breast cancer remains unclear. Therefore, this study investigated the relationship between serum reproductive hormone concentrations and patient characteristics and hormone receptor status among postmenopausal Chinese women with breast cancer.

**Materials and Methods:**

The medical records of 352 postmenopausal breast cancer patients who underwent an operation between October 2007 and October 2010 at the Department of Breast Tumor Surgery of Zhejiang Cancer Hospital were retrospectively evaluated. Serum levels of reproductive hormones were measured before surgery by liquid-chromatography tandem mass spectrometry. Hormone receptor levels were measured by an immunohistochemical assay using a mouse monoclonal antibody. The associations between serum hormone concentrations and hormone receptors were investigated by analysis of covariance.

**Results:**

In this patient cohort, the serum level of luteinizing hormone (LH) declined with PMP duration. The median LH concentration was significantly higher in patients within 5 years of PMP than that in patients with PMP duration exceeding 5 years (23 vs. 18.32 mIU/ml, *P* <.0001). Significantly more patients with strong estrogen receptor (ER) or progesterone receptor (PR) expression had postmenopausal durations of less than 5 years compared to those with postmenopausal durations greater than 5 years (103 vs. 61 cases, *P* = .019; 93 vs. 46 cases, *P* = .0005). While most patients either lacked (97.1%) or co-expressed (84.3%) ER and PR, some patients expressed either ER or PR alone. ER and PR expression were negatively associated with receptor-tyrosine kinase erbB-2 (HER2) expression in postmenopausal patients with breast cancer. Meanwhile, increased ER and PR expression were associated with decreased serum levels of LH or follicle-stimulating hormone (FSH).

**Conclusion:**

Decreased serum LH and FSH levels were associated with increased ER and PR expressions and decreased HER2 expression in postmenopausal patients with breast cancer.

## Introduction

Breast cancer is one of the most common cancers in women worldwide, with an annually increasing incidence (1, 2). In 2020, 276,480 new patients with breast cancer and 42,170 deaths occurred in the United States (3). However, in China, the incidence rate of breast cancer is more than twice that of the world, and the incidence of elderly patients with breast cancer is also rising (4). The increase is related to changes in reproductive patterns and the use of mammography screening. Meanwhile, the trend is also associated with physical inactivity, lifestyle changes, menopausal hormone use, and the prevalence of obesity (5).

Breast cancer is a unique disease with multiple clinical subtypes. However, its pathogenesis remains unclear (6). De Waard first proposed the presence of two pathways and age-specific incidence rate curves in breast cancer (7, 8). In one pathway, the incidence of premenopausal breast cancer peaks early in life, while the peak incidence of postmenopausal disease in the second pathway occurs later in life. The two peak ages reported in China were 45–55 and 70–74 years, respectively (9).

While the causes leading to the development of breast cancer are unknown, the associated risk factors include age and elements related to reproductive life (10). Among those risk factors, hormones such as estrogen (E) and progesterone (P) play an important role in accelerating breast cancer cell growth. Furthermore, the cumulative exposure to hormones such as E and P also increases the likelihood of breast cancer (11–13).

The serum estrogens include estrone (E1), estradiol (E2), and estriol (E3). Among these estrogens, E2 affects cell proliferation and apoptosis by interacting with the estrogen receptor (ER) in breast tissue, thus affecting the development and progression of breast cancer (14). Furthermore, E2 levels were positively correlated with the risk of postmenopausal breast cancer (15–17).

Breast cancer cells express the ER or progesterone receptor (PR), and about two-thirds of breast cancers are ER-positive and/or PR-positive (18). Breast cancer cells expressing ER or PR require E or P to grow. In addition to ER and PR, receptor tyrosine-protein kinase erbB-2 (HER2) plays a crucial role in breast cancer progression (19). Cancer cells with increased HER2 expression tend to grow and spread more aggressively compared to cancer cells lacking HER2 expression (20). Clinically, the positivity status of ER, PR, or HER2 alone or in combination is critically involved in the selection of therapeutic approaches and determines patient outcomes in breast cancer (21, 22).

Like other types of cancer, the incidence of breast cancer increases with age. Approximately two-thirds of invasive breast cancers occur in women aged 55 years or older; thus, most patients with breast cancer are postmenopausal. However, whether postmenopausal duration affects hormone levels and hormone receptor expression remains unknown. This study measured serum levels of reproductive hormones, namely, luteinizing hormone (LH), E2, P, testosterone (T), follicle-stimulating hormone (FSH), and prolactin (PRL) in postmenopausal patients with breast cancer. The expression levels of ER, PR, HER2, and p53 were also determined. Furthermore, the relationships between these receptors and hormones were evaluated.

## Materials and Methods

This study included 352 postmenopausal patients diagnosed with breast cancer. The diagnosis was made on the basis of pathological findings from thick-needle biopsy specimens of breast tissues and included invasive ductal carcinoma *in situ* (DCIS). We retrospectively extracted and analyzed data from the patient database of this cohort from October 2007 to October 2010. These data were gathered prior to undergoing chemotherapy, radiotherapy, or hormone therapy. No patients had chronic hepatitis or nephritis, and all had normal liver and renal functions.

This study was reviewed and approved by the Medical Ethics Committee of Zhejiang Cancer Hospital of China. The patients provided their written informed consent.

### Serum Hormonal Levels

The serum concentrations of six hormones (LH, E2, T, P, FSH, and PRL) were assayed before the patients underwent surgery. The concentrations were measured by chemiluminescence immunoassay (CLIA) on a UniCel DxI 800 system (Beckman Coulter, Brea, CA, USA) following the instructions of the manufacturer for routine laboratory tests. The laboratory standard values for these hormones were 7.7–58.5 mIU/ml for LH, 10–40 pg/ml for E2, 0–.78 pg/ml for P, 0–0.75 pg/ml for T, 25.8–113.59 pg/ml for FSH, and 0–19.64 pg/ml for PRL.

### Determination of Hormone Receptor Status

Less than 30 min after surgery, the tumor specimens were fixed with 4% neutral formalin and embedded in paraffin blocks. The blocks were subsequently sliced into 4-μm sections. Immunohistochemistry was performed using a Universal DAB Detection kit (Ventana Medical Systems, Tucson, AZ, USA) following the instructions of the manufacturer. Anti-ER (clone No. SPl) and anti-PR (clone No. PgR636) monoclonal antibodies were purchased from Dako, Inc. (Carpinteria, CA, USA), and an anti-HER2 monoclonal antibody (clone No. 4B5) was also used. The expressions of P53 and TOPO II in the study were assessed using monoclonal anti-P53 antibody (cat.nos: ab131442) and anti-TOPO II antibody (clone KISI-DAKO, UK/DN dilution 1:50).

**Figure 1:**
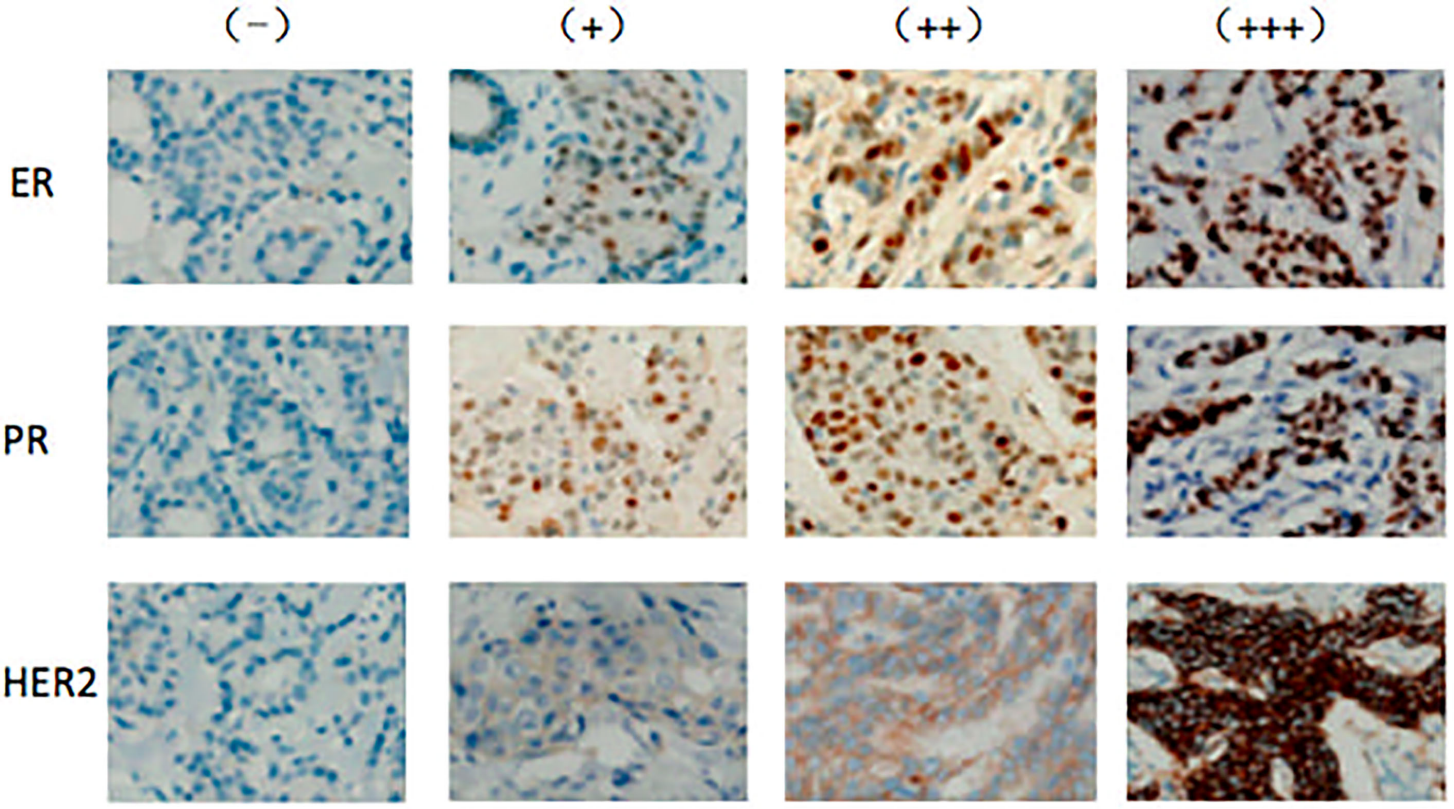
Expression of ER, PR, and HER2 in PMP patients with breast cancer Expression levels of ER, PR, and HER2 were determined by immunohistochemistry and assessed by staining intensity from no expression (−) to weak (+), intermediate (++), and strong (+++) staining.

ER and PR staining was assessed as described in the “Immunohistochemistry guide for the staining of estrogen and progesterone receptor in breast cancer (2015 edition in Chinese),” with a slight modification (**Figure 1**). ER or PR positivity or negativity was defined according to the percentages of nuclear-stained cells among all tumor cells in the entire section. Cases with percentages above 10% were defined as positive. If nuclear staining was absent or the percentage of nuclear-staining cells was less than 10%, the case was defined as negative. The ER and PR-positive cases were further divided into three groups based on nuclear staining intensity as weak (pale-yellow, +), intermediate (brown-yellow, ++), and strong (dark-brown, +++).

HER2 staining was assessed as described in the “Testing guide for the staining of HER2 in breast cancer (2014 edition in Chinese).” HER2 staining in each case was scored as 0, +, ++, or +++ (**Figure 1**). A (0) score indicated no or incomplete and weak membrane staining in ≤10% of cancer cells. A (+) score indicated an incomplete and weak membrane staining in > 10% of cancer cells. A (++) score indicated an incomplete and/or weak to moderate membrane staining in >10% of cancer cells or strong and complete membrane staining in ≤10% of cancer cells. A (+++) score was defined as strong and complete membrane staining in >10% of cancer cells. HER2 was considered negative or positive for scores of (0)/(+) and (+++), respectively. A (++) HER2 score was defined as uncertain, and further tests such as fluorescence *in situ* hybridization (FISH) or genotyping were required to confirm positivity.

The scoring and intensity of P53 were evaluated using light microscopy (100*) according to the methods of Papamistou et al. (23). So, we characterized P53 expression as negative, weak, and strong.

The TOPO II protein expression levels were assessed by measuring the corresponding staining intensity levels provided by digital image analysis. Using normal epithelia as the control group, we characterized TOPO II expression as negative, weak, and strong.

### Statistical Analysis

In the analysis section, IBM statistics could be cited as IBM Corp. Released 2010. IBM SPSS Statistics for Windows, Version 19.0. Armonk, NY: IBM Corp.

## Results

The characteristics of 352 postmenopausal patients with breast cancer who were enrolled in this study are summarized in **Table 1**. The median age of this cohort was 57 years, with most (58.81%) patients aged 50–59 years; 1.99 and 11.36% were aged below 50 or above 70 years, respectively. Pathologically, 278 of the 352 (78.98%) patients had invasive ductal carcinoma, a dominant type of breast cancer. In this cohort, 58.24 and 48.01% of patients expressed ER and PR, respectively. Most patients (76.7%) were HER2-positive.

**Table 1:** Patients’ characteristics.

Characteristics	Variance	N	%
Age (years)	40–49	7	1.99
	50–59	207	58.81
	60–69	98	27.84
	70 above	40	11.36
Pathology	Invasive ductal	278	78.98
	Others	74	21.02
ER expression	+++	124	35.23
	++	40	11.36
	+	30	8.52
	−	147	41.76
PR expression	+++	63	17.90
	++	54	15.34
	+	41	11.65
	−	183	51.99
HER2 expression	+++	77	21.88
	++	100	28.41
	+	72	20.45
	−	82	23.30

ER, estrogen receptor; PR, progesterone receptor; HER2, receptor-tyrosine kinase erbB-2.

We first measured the serum levels of six hormones (LH, E2, T, P, FSH, and PRL) in postmenopausal patients with breast cancer by CLIA. We arbitrarily divided the patient cohort into four groups based on postmenopausal duration (1–5, 6–10, 11–20, and more than 21 years). The median values and ranges of each hormone are listed in **Table 2**. Serum levels of E2 and P were less than 10 pg/ml and 0.1 ng/ml, respectively, with undetectable values in 66.9% (228 of 341) and 22.6% (77 of 341) of the patients, respectively.

**Table 2:** Association of PMP years with hormones in breast cancer patients.

PMP years		Hormones					
		LH	E2	P	T	FSH	PRL
1–5	N	154	55	123	152	154	153
	Median	23	13.5	0.1	0.44	54.98	13.85
	Range	3.56–57.8	11–120	0.1–1.3	0.08–4.7	3.2–138	3.52–89.6
6–10	N	77	26	62	77	77	77
	Median	19.48	12	0.1	0.42	54.7	12.23
	Range	6.13–50.2	11–22	0.1–0.6	0.13–1.2	0.86–150	4.51–114
11–20	N	81	26	59	81	81	81
	Median	17.36	13	0.1	0.46	50.97	11.84
	Range	1.16–46.1	11–36	0.1–0.7	0.08–1.4	5.6–150	0.03–161
≥21	N	29	6	20	29	29	29
	Median	16.87	10.5	0.1	0.39	54.38	12.67
	Range	8.01–32.4	11–17	0.1–0.8	0.08–1.15	27.92–150	6.3–34.32
P		0.0001*	<0.05	<0.05	<0.05	<0.05	<0.05

PMP, postmenopausal; LH, luteinizing hormone; E2, estradiol; P, progesterone; T, testosterone; FSH, follicle-stimulating hormone; PRL, prolactin.

*Comparison between group of PMP 1–5 and group of PMP 6 above.

Among the six hormones, the serum level of LH declined with postmenopausal duration in this patient cohort. The median LH value was significantly higher in patients with a postmenopausal duration of 5 years or less (23 mIU/ml) than in patients with a postmenopausal duration exceeding 5 years (18.32 mIU/ml, *P* <.0001). The difference between the postmenopausal duration of 1–5 years and the postmenopausal duration of 11–20 or ≥21 years was also significant. However, a significant difference in the serum levels of other hormones in patients with different postmenopausal durations was not observed.

We subsequently determined hormone receptor (ER, PR, and HER2) expression by immunohistochemistry. As shown in **Figure 1**, the expression levels of these hormone receptors varied from negative to weak positive (+) and strong positive (++ and +++ combined). To simplify the analysis, we arbitrarily divided the patient cohort into two groups based on postmenopausal duration (1–5 and ≥6 years). The numbers of patients expressing variable levels of hormone receptors are listed in **Table 3**. The number of women with strong ER and PR expression was significantly higher in patients with a postmenopausal duration exceeding 5 years (103 and 93 cases, respectively) than in patients with a postmenopausal duration of up to 5 years (61 and 46 cases, *P* = .019 and *P* = .0005, respectively). No differences in HER2 expression levels in breast cancer patients according to postmenopausal duration were observed.

**Table 3:** Association between PMP and hormone receptors in breast cancer patients.

Hormones receptors		PMP (years)		P
		1–5 (n)	6 above (n)	
ER expression	Negative	74	73	0.019*
	Weak positive	19	11
	Strong positive	61	103
PR expression	Negative	89	94	0.005*
	Weak positive	19	32
	Strong positive	46	93
HER2 expression	Negative	32	50	0.091
	Weak positive	30	42
	Strong positive	89	88
P53 expression	Negative	56	78	0.542
	Weak positive	37	42
	Strong positive	44	52
TOPO II expression	Negative	16	32	0.284
	Weak positive	47	51
	Strong positive	6	6

PMP, postmenopausal; ER, estrogen receptor; PR, progesterone receptor; HER2, receptor-tyrosine kinase erbB-2; TOPO II, topoisomerase II.

*Comparison between group of negative and group of strong positive.

In addition to hormone receptors, we measured the expression of tumor proteins p53 and type II topoisomerase in this patient cohort and found no differences in the expression among postmenopausal patients with breast cancer.

We subsequently analyzed the association between hormones and hormone receptors in postmenopausal patients with breast cancer. As shown in **Table 4**, patients with strong tumor ER or PR expression (++ and +++ combined) had significantly reduced serum LH levels (18.6 and 19.1 mIU/ml, respectively) compared to those in patients without tumor ER or PR expression (21.7 and 21 mIU/ml, *P* = .001 and.008, respectively). As well, the levels of FSH (52.415 and 51.39 pg/ml, respectively) in patients with strong tumor ER or PR expression were lower than those in patients without tumor ER or PR expression (55.6 and 53.9 pg/ml, P = 0.03 and P = 0.031, respectively). In contrast, patients with strong tumor HER2 expression had significantly elevated serum LH levels (22.1 mIU/ml) compared to those in patients without tumor HER2 expression (18.3 mIU/ml, *P* = .011). No associations between p53 expression and serum levels of any hormone were observed in postmenopausal patients with breast cancer.

**Table 4:** Association between hormones and hormone receptors.

Receptors		Hormones											
		LH	p	E2	p	P	p	T	p	FSH	p	PRL	p
ER expression	Negative	21.7	0.001	13	0.99	0.1	0.38	0.41	0.06	55.6	0.03	13.42	0.37
	Weak postive	21		15		0.2		0.42		54.84		14.17	
	Strong positive	18.63		12		0.1		0.44		52.415		12.6	
PR expression	Negative	20.99	0.008	10	0.52	0.1	0.72	0.405	0.015	53.9	0.031	13.54	0.65
	Weak postive	20.03		10		0.2		0.37		13.0		14.71	
	Strong positive	19.05		10		0.1		0.49		51.39		11.55	
HER 2 expression	Negative	18.33	0.011	14	0.011	0.1	0.99	0.44	0.93	51.25	0.07	11.62	0.18
	Weak postive	17.59		13		0.1		0.37		49.53		13.32	
	Strong positive	22.05		12		0.1		0.43		58.44		13.525	
P53 expression	Negative	20.08	0.20	13	0.87	0.1	0.80	0.425	0.58	54.34	0.19	13.42	0.79
	Weak postive	18.73		12		0.1		0.49		51.86		12.23	
	Strong positive	20.79		12		0.1		0.40		53.82		12.32	

ER, estrogen receptor; PR, progesterone receptor; HER2, receptor-tyrosine kinase erbB-2; LH, luteinizing hormone; E2, estradiol; P, progesterone; T, testosterone; FSH, follicle-stimulating hormone; PRL, prolactin.

p Comparison between group of negative and group of strong positive.

A chi-square analysis to evaluate the association between these three hormone receptors in postmenopausal patients with breast cancer showed a strong positive association between ER and PR expression in this patient cohort. As shown in **Table 5**, the number of patients who were double-negative or double-positive for ER and PR was significantly higher than those of patients with single-negative or -positive ER or PR status. Among 173 patients without an ER expression, 168 (97.1%) cases also lacked PR expression. However, 84.3% (54 of 64) of the patients with a strong PR expression (+++) also had a strong ER expression.

**Table 5:** Association between any two hormones receptors.

Hormones receptors		ER					HER2				
		−	+	++	+++	P	−	+	++	+++	p
PR	−	168	13	11	16	0.001*	34	36	53	59	0.001*
	+	5	10	9	20		12	11	11	8	
	++	0	5	16	36	0.001#	16	11	21	7	0.001#
	+++	0	3	7	54		24	16	7	4	
HER2	−	28	11	7	40	0.001*					
	+	28	6	8	33						
	++	38	7	20	39	0.022#					
	+++	52	7	7	13						

*Comparison between (−) group and (+++) group, #Comparison between (−) group and (++/+++) combined group.

In contrast, we observed a negative association between the expression of HER2 and ER or PR in postmenopausal patients with breast cancer. Patients without ER or PR expression tended to have a strong HER2 expression and vice versa. Among the 146 and 182 patients without ER and PR expression, 90 (61.6%) and 112 (61.5%) cases had strong ER expression (++/+++), respectively. In contrast, only 13 (16.5) and 4 (5.1%) of 79 patients with strong HER2 expression (+++) had strong ER or PR expression, respectively.

## Discussion

Circulating reproductive hormones play an important role in breast cancer development and progression. Long-term exposure to high amounts of E in the blood increases the risk of breast cancer (24, 25). Binding to receptors, hormones accelerate breast cell proliferation. A previous study showed that higher circulating E1 and E2 increased the incidence of breast cancer (26). Furthermore, the levels of E2 in postmenopausal Chinese patients with breast cancer were higher than those of healthy subjects (27). This study focused on postmenopausal patients to explore the association between hormones and hormone receptors since a menstrual cycle-mediated variation in serum hormone levels is minimized in postmenopausal patients. To our knowledge, this is the first study to comprehensively measure the levels of reproductive hormones and receptors in a relatively large cohort of postmenopausal patients with breast cancer.

LH, FSH, and PRL are produced by the pituitary gland and affect the ovaries. In FSH stimulation of ovarian follicles to produce estrogen, whereas LH stimulates the corpus luteum to secrete P in premenopausal women. With ovarian atrophy, the production of E and P drops with increasing postmenopausal duration. In this study, the serum levels of LH, FSH, and PRL decreased in the years following menopause, although their levels remained stable in patients who had experienced postmenopausal more than 21 years ago. While some postmenopausal patients maintained normal E2 and T levels, a significant number of patients (66.9% for E2 and 22.6% for T) had undetectable concentrations of these hormones, suggesting a clinical indication for E therapy in this patient population.

ER and PR are the most widely studied markers in breast cancer (28), and their expression levels are used as predictive markers of response to endocrine therapy (29). This study assessed the effects of postmenopausal on ER and PR expression in breast cancer and found that ER and PR expression were significantly associated with postmenopausal duration. Considering the inhibitory effect of the hormones on ER and PR expression, the enhanced expression may be the result of a decline in E and P levels in postmenopausal patients.

ER and PR expression were significantly correlated with postmenopausal patients with breast cancer. The number of patients with double-negative or double-positive ER and PR expression was significantly higher than those with single-negative ER and PR expression. We observed patients with single-positive ER or PR expression. Nadji et al. analyzed 5,993 patients with breast cancer and found no cases that were ER-negative but PR-positive (30). In contrast, other studies have reported independent expression of ER and PR (31, 32), consistent with our findings.

It is unclear whether hormone levels correlate with ER or PR expression in breast cancer patients. In this cohort, we observed a positive correlation between serum hormone levels and ER or PR expression, consistent with previous reports of the inhibitory effect of estrogen on ER or PR expression (33). In contrast, the hormone levels were higher in HER2-positive patients than in HER2-negative patients, supporting the observed negative association between ER or PR and HER2 expression.

Some limitations of this study should be considered when interpreting these results. First, this retrospective study involved patients from a single center. Second, we evaluated only the relationship between serum reproductive hormone concentration and patient characteristics and hormone receptor status among post-menopausal Chinese women; the survival outcomes were not considered. Finally, records of patients were partly dismissed. Therefore, these results should be regarded as preliminary, and additional prospective, randomized, large-sample, multi-center phase III clinical trials must confirm our findings.

### Conclusion

The results of this study showed decreased serum hormone levels over the postmenopausal course in patients with breast cancer. The number of patients with strong ER and PR or negative HER2 expression increased in the later years compared with the early years of PMP. While most patients either lacked or co-expressed ER and PR, some patients expressed either ER or PR alone. ER and PR expression were negatively associated with HER2 expression in postmenopausal patients with breast cancer. Increased ER and PR expression was associated with decreased serum LH or FSH levels. These results indicated that postmenopausal-mediated decreases in serum LH and FSH levels were associated with increased ER and PR expression and decreased HER2 expression in patients with breast cancer. Overall, the present findings provide an improved understanding of the association between hormones and receptors in postmenopausal patients with breast cancer.

## Data Availability Statement

The raw data supporting the conclusions of this article will be made available by the authors, without undue reservation.

## Ethics Statement

The studies involving human participants were reviewed and approved by the Medical Ethics Committee of Zhejiang Cancer Hospital of China. The patients/participants provided their written informed consent to participate in this study. Written informed consent was obtained from the individual(s) for the publication of any potentially identifiable images or data included in this article.

## Author Contributions

FW and DZ contributed to conception and design of the study. CJ, PW, XH, XD, XX, and JN organized database. CJ wrote the first draft of the manuscript. CJ, FW, and DZ wrote sections of the manuscript. All authors listed have made a substantial, direct, and intellectual contribution to the work and approved it for publication.

## Funding

The authors declare that this study received funding from the Medical and Health Science and Technology Program of Zhejiang Province (Nos. 2020KY084, 2019KY041, 2011RCA014, 2006A016, and 2005B012), the National Natural Science Foundation of Zhejiang Province of China (LY15H1800012015), and the National Natural Science Foundation of China (Nos. 81502646 and 81502647).

## Conflict of Interest

The authors declare that the research was conducted in the absence of any commercial or financial relationships that could be construed as a potential conflict of interest.

## Publisher’s Note

All claims expressed in this article are solely those of the authors and do not necessarily represent those of their affiliated organizations, or those of the publisher, the editors and the reviewers. Any product that may be evaluated in this article, or claim that may be made by its manufacturer, is not guaranteed or endorsed by the publisher.

## Abbreviations

PMP, postmenopausal; LH, luteinizing hormone; ER, estrogen receptor; PR, progesterone receptor; HER2, receptor-tyrosine kinase erbB-2; FSH, follicle-stimulating hormone; DCIS, ductal carcinoma *in situ*; E, estrogen; E2, estradiol; P, progesterone; T, testosterone; PRL, prolactin; FISH, fluorescence *in situ* hybridization.
